# VascX Models: Deep Ensembles for Retinal Vascular Analysis From Color Fundus Images

**DOI:** 10.1167/tvst.14.7.19

**Published:** 2025-07-23

**Authors:** Jose Vargas Quiros, Bart Liefers, Karin A. van Garderen, Jeroen P. Vermeulen, Caroline Klaver

**Affiliations:** 1 Department of Ophthalmology, Erasmus University Medical Center, Rotterdam, the Netherlands; 2 Department of Epidemiology, Erasmus University Medical Center, Rotterdam, the Netherlands; 3 Department of Ophthalmology, Radboud University Medical Center, Nijmegen, the Netherlands; 4 Institute of Molecular and Clinical Ophthalmology, University of Basel, Basel, Switzerland

**Keywords:** VascX, vascular biomarkers, vascular features, medical image analysis, fundus segmentation, artery-vein segmentation, optic disc segmentation, fovea detection

## Abstract

**Purpose:**

To present and validate deep learning model ensembles (VascX) for vessel, artery-vein, optic disc segmentation, and fovea localization for color fundus images (CFIs). VascX preprocessing and inference code and model weights were made publicly available to facilitate research on retinal vasculature.

**Methods:**

For model training, we combined over 15 published annotated datasets with CFIs from Dutch studies (mainly the Rotterdam Study). This resulted in diverse development sets with a variety of patient characteristics and imaging conditions. We trained UNet model ensembles using a new, more robust preprocessing algorithm and strong data augmentations. We compared VascX segmentation performance (Dice) to models with publicly available weights: AutoMorph and LittleWNet. We compared the quality of VascX (and previous models’) features by measuring agreement (mean absolute error [MAE] and Pearson correlation) with features extracted from grader segmentations.

**Results:**

Dice scores revealed better performance from VascX across most datasets evaluated, especially for artery-vein and optic disc segmentation. VascX performed more consistently as the quality of the images decreased and for both disc and fovea-centered images. These improvements translated into higher-quality vascular features. Of 24 features evaluated, 14 showed a significant improvement in MAE when compared to AutoMorph and 23 when compared to LWNet. VascX had the highest correlations with ground-truth features in all but two cases.

**Conclusions:**

VascX models perform well across a variety of conditions, likely due to the size and diversity of our development sets. VascX represents an important improvement in segmentation quality that translates into better vascular features to support more robust analyses of the retinal vasculature.

**Translational Relevance:**

By making VascX public, we aim to facilitate and improve research linking retinal vascular biomarkers to ophthalmic and systemic conditions, relevant for the detection, prevention, and monitoring of disease.

## Introduction

Interest in measuring retinal calibers dates back to the 1970s, when Parr and Spears^1^ published their approach to approximate the caliber of the central retinal artery and vein using the calibers of their largest branches. Since the late 1990s, various semiautomatic methods have been developed to perform these calculations with different levels of human and machine involvement, enhancing the efficiency of such measurements.^2^ Among these developments were prominent tools such as SIVA (Singapore I Vessel Assessment),^3^^–^^7^ IVAN,^8^^,^^9^ and VAMPIRE (Vascular Assessment and Measurement Platform for Images of the Retina).^10^^–^^12^

Today, the understanding of retinal vasculature and its association with diseases has expanded substantially. A significant focus has been placed on biomarkers such as retinal calibers, tortuosity, and branching characteristics. These biomarkers are now studied in relation not only to ocular diseases but also to systemic conditions, particularly cardiovascular diseases.^13^^–^^15^^,^^59^ More recently, attention has turned to the retina as a window into brain pathology, with studies linking retinal vascular changes to neurodegenerative diseases such as Alzheimer disease.^16^ Previous work has shown that automatically extracted vascular features improve the performance of diabetic retinopathy (DR) detection and classification (Abtahi M, et al. *IOVS.* 2024;65:ARVO E-Abstract PB0060).

Parallel to the scientific advances, deep learning brought about groundbreaking improvements in segmentation quality across a variety of tasks.^17^ This triggered the development of multiple datasets, deep learning architectures, and models for the segmentation of the main anatomic structures visible in color fundus images (CFIs): vessel segmentation,^18^^,^^19^ artery-vein segmentation,^20^^,^^21^ and disc segmentation. The task of assessing fundus image quality has also received attention.^22^^–^^24^ More recent are efforts to integrate these models into pipelines for fully automated, explainable, vascular feature extraction. AutoMorph is the most notable example of an open-source deep learning–based pipeline integrating vessel segmentation, artery-vein segmentation, disc segmentation, and vascular feature extraction.^25^ AutoMorph has been applied in various studies, including on the reproducibility of its features.^26^^–^^29^ The availability of disc segmentation models in AutoMorph allows for region-based analysis, which isolates features from specific areas of the retina.

Despite these developments, the robust segmentation of retinal morphology (arteries, veins, optic disc, fovea) from CFIs across a variety of patient and imaging conditions remains unsolved. Most publicly available training datasets for artery-vein segmentation are small and homogeneous, often limited to images from a single device, centered on a single anatomic region (fovea or optic disc), and with a fixed field of view. Until recently,^30^ the largest publicly available dataset for artery-vein segmentation contained only 45 images.^31^ Additionally, the pathology and demographics of the study population are often restricted, which significantly impacts the generalizability of the models trained on these datasets. This affects the downstream extraction of explainable vascular features, which depend on the quality of such segmentations^29^ (Somfai GM, et al. *IOVS*. 2023;64:ARVO E-Abstract PB0038). This is particularly problematic in analyses of large cohort studies and clinical data, which may encompass a diverse range of devices and capture conditions. To overcome these drawbacks, larger and more diverse training datasets are needed to develop models that work reliably across a broader spectrum of conditions.

In this article, we present contributions toward a robust analysis of the retinal vasculature through improved segmentation and localization models. Our contributions are the following:

1. We present models for optic disc, vessel, and artery-vein segmentation and fovea localization, fundamental models in the development of CFI analysis systems, especially of the retinal vasculature. We built our models using public datasets and new annotations by professional graders on diverse sets of CFIs from Dutch studies, mainly the Rotterdam Study.
2. We benchmark our models against publicly available models^25^^,^^20^ and show significant improvements in segmentation performance. We characterize the performance of the models with respect to image quality as measured using a quality assessment model.
3. We evaluate the quality of features extracted from model output by comparing them to features extracted from segmentations made by experienced graders.
4. We publicly release and make open-source our preprocessing and inference code, including model weights.

## Methods

VascX models include vessel, artery-vein (A/V), and disc segmentation and fovea localization model ensembles. Model weights and inference code are publicly available at https://github.com/Eyened/rtnls_vascx_models. This section details the development of these model ensembles, including source datasets, annotations, and model training.

### Datasets

We trained and evaluated our models on a combination of public datasets and images from the Rotterdam Study (RS) and other Dutch studies.

#### Public Datasets

We collected publicly available datasets from previous work. These datasets have different patient demographics, pathologies, retinal regions (fovea and disc centered), field of view, and countries of origin. Table 1 shows an overview of these datasets. For complementary details, including capture devices and resolution, see Supplementary Table S5.

**Table 1.** Overview of Datasets Used in This Work for Training and/or Evaluation of Various Segmentation and Localization Tasks

Dataset	*N*	Origin	Ages Pathology		Region	FOV
Vessel Segmentation						
Chase DB^32^	28	UK	Children	—	OD	35°
DRHAGIS^33^	40	UK	NR	10 GC, 10 HT, 10 DR, 10 AMD	M	45°
HRF^31^	45	GER	NR	15 H, 15 DR, 15 GC	M	45°
RETA^34^	54	IND	NR	Signs of DR	M	50°
FIVES^35^	800	CN	4–83	H, GC, DR, AMD	M	50°
Leuven-Haifa^30^	240	BE	18–90	75 NTG, 63 HTG, 56 H, 30 O	OD	30°
Rotterdam (ours)	352	NL	40+	See Figure 1	
Total	**1559**				
Artery vein segmentation						
RITE^36^	40	NL	25–90	7 DR, 33 no DR	M	45°
HRF-AV^37^	45	GER	NR	15 H, 15 DR, 15 GC	M	45°
Les-AV^38^	22	NR	NR	NR	OD	30°
IOSTAR^39^	30	NR	NR	NR	M, OD	45°
AVRDB^40^	100	PAK	25–80	80 HT, 20 H	M, OD	45°
Leuven-Haifa^30^	240	BE	18–90	75 NTG, 63 HTG, 56 H, 30 O	OD	30°
Rotterdam (ours)	215	NL	40+	See Figure 1	
Total	**562**				
Disc segmentation						
ORIGA^41^	650	SGP	40–80	Multiple	OD	NR
PAPILA^42^	488	SPN	15–90	87 GC, 333 H, 68 O	OD	30°
IDRiD^43^	81	IND	NR	Signs of DR	M	50°
ADAM^44^	821^a^	CN	53*.*2 ± 15*.*6	267 AMD, 933 O	M, OD, MP	45°
PALM^45^	1179^b^	CN	37*.*5 ± 15*.*91	637 PM, 563 O	M, OD, MP	45°°
REFUGE2^46^	2000	CN	NR	280 GC, 1720 H/O	M, OD, MP	45°
Rotterdam (ours)	1225	NL	40+	See Figure 1		
Total	**7464**					
Fovea localization						
IDRiD^43^	516	IND	NR	Signs of DR	M	50°
ADAM^44^	1200	CN	53*.*2 ± 15*.*6	267 AMD, 933 O	M, OD, MP	45°
PALM^45^	1200	CN	37*.*5 ± 15*.*91	637 PM, 563 O	M, OD, MP	45°
REFUGE2^46^	2000	CN	NR	280 GC, 1720 H/O	M, OD, MP	45°
Rotterdam (ours)	10,908	NL	40+	See Figure 1		
Total	**15** **,** **824**					

BE, Belgium; CN, China; FOV, field of view; GC, glaucoma (all types); GER, Germany; H, healthy; HT, hypertension; HTG, high-tension glaucoma; IND, India; M, macula-centered CFI; MP, CFI centered between macula and optic disc; NL, The Netherlands; NR, not reported in the source manuscript; NTG, normal-tension glaucoma; O, other eye diseases; OD, optic disc centered CFI; PM, pathological myopia; SGP, Singapore; SPN, Spain; UK, United Kingdom.

a In total, 379 of 1200 samples were discarded due to containing no disc segmentation for partially visible discs.

b In total, 21 of 1200 CFIs were discarded due to having no associated annotation.

We included several well-known vessel and artery-vein segmentation datasets with patients with DR.^31^^,^^33^^,^^34^^,^^37^ We excluded the DRIVE dataset^36^^,^^47^ due to its low resolution (584 × 565 pixels). We included the larger and more recent FIVES^35^ and Leuven-Haifa^30^ datasets, which contain a mixture of diseased and healthy patients. On the other hand, most datasets with disc segmentations were originally collected for the assessment of glaucoma.^41^^,^^42^^,^^46^ The REFUGE2 dataset, an extension of the original REFUGE dataset,^48^ is notable for its size (2000 CFIs), the various anatomic regions it captures, and its inclusion of fovea location annotations. We also included ADAM (age-related macular degeneration),^44^ PALM (pathological myopia),^45^ and IDRiD (diabetic retinopathy),^43^ which also contain fovea positions. The field of view of the images in our training datasets is between 30° and 50°, which includes most standard fundus imaging devices. We excluded datasets with widefield and ultra-widefield CFIs due to the potential for significant domain shift.

Finally, we used the Eye-Quality (EyeQ) dataset (Table 1) for the quality estimation algorithm. This dataset contains manual gradings of fundus quality of 28,792 CFIs from the EyePACS Diabetic Retinopathy dataset.^49^ CFIs were graded using a three-class system as unusable/bad, usable, and good quality. This dataset is unique for its size and for containing a variety of capture devices and image conditions. For this reason, we did not augment this dataset with our own annotations.

#### Rotterdam Datasets

We complemented the CFIs from the public datasets with images available to the Department of Ophthalmology at Erasmus Medical Center in Rotterdam, mainly the RS. The RS is a prospective population-based cohort study of people living in Ommoord, a district of the city of Rotterdam.^50^ The RS consists of four cohorts, all of which were used in this work. The minimum age of study participants varies between *>*55 in the first cohort and *>*40 in the fourth. Each cohort was followed for multiple rounds of follow-up examinations every 4 to 5 years. Most of the visits in the RS involved the capture of CFIs on both eyes. Due to the multidecade span of the RS, multiple devices, capture conditions, and fields (macula and disc centered) are present in the dataset.

In addition to the RS, we included images captured during the Randomized Controlled Trial for Age-Related Macular Degeneration (AMD-Life),^51^ which consisted of 150 patients aged 55 to 85 years imaged with a Topcon 3D OCT 2000-plus (Tokyo, Japan) and Topcon TRC-50EX cameras. Images from the Dutch Myopia Study (MYST),^52^ which included younger subjects (mean [SD] age of 47.3 [12.9] years), were also used. These images were captured with a Topcon TRC-50EX.

#### CFI Selection and Weighing

We used most public datasets in their entirety without filtering, after harmonizing annotations when necessary. The main exception was disc segmentations in the ADAM dataset, where 379 of 1200 samples were discarded due to containing no disc segmentation for partially visible discs.

Regarding the Rotterdam sets, we sampled CFIs at random from all available images. We included fundus images captured by optical coherence tomography (OCT) machines to improve the diversity of devices in the dataset. For the vessel and A/V segmentation datasets (Table 1), due to the much larger size of the Rotterdam Study compared to AMD-Life and MYST, we oversampled CFIs from these sets to make up 15% of the samples. We asked graders to exclude images for which they found the quality to be completely unusable for the task at hand due to overall low visibility of the vessels. However, we were intentionally inclusive regarding quality to encourage a more diverse and challenging development set. We included images considered *unusable* by the AutoMorph quality estimation algorithm, under the consideration that although the general quality level of these images is poor, many contained clearly usable vascular information for measuring biomarkers. Examples can be found on the main vessel arches (e.g., central retinal equivalents [CREs] or opening angles).

For disc segmentation, due to the large size of publicly available datasets (Table 1), we first used a model trained on publicly available datasets to automatically identify failure or challenging CFIs. We selected CFIs for which no disc was detected or a disconnected disc mask was produced by the model. A trained grader reviewed these images and identified the ones where the disc was present but incorrectly segmented, and these were included in the training set. In practice, these included many analog, macula-centered images from the Rotterdam sets, which especially challenged the model.

**Figure 1.**
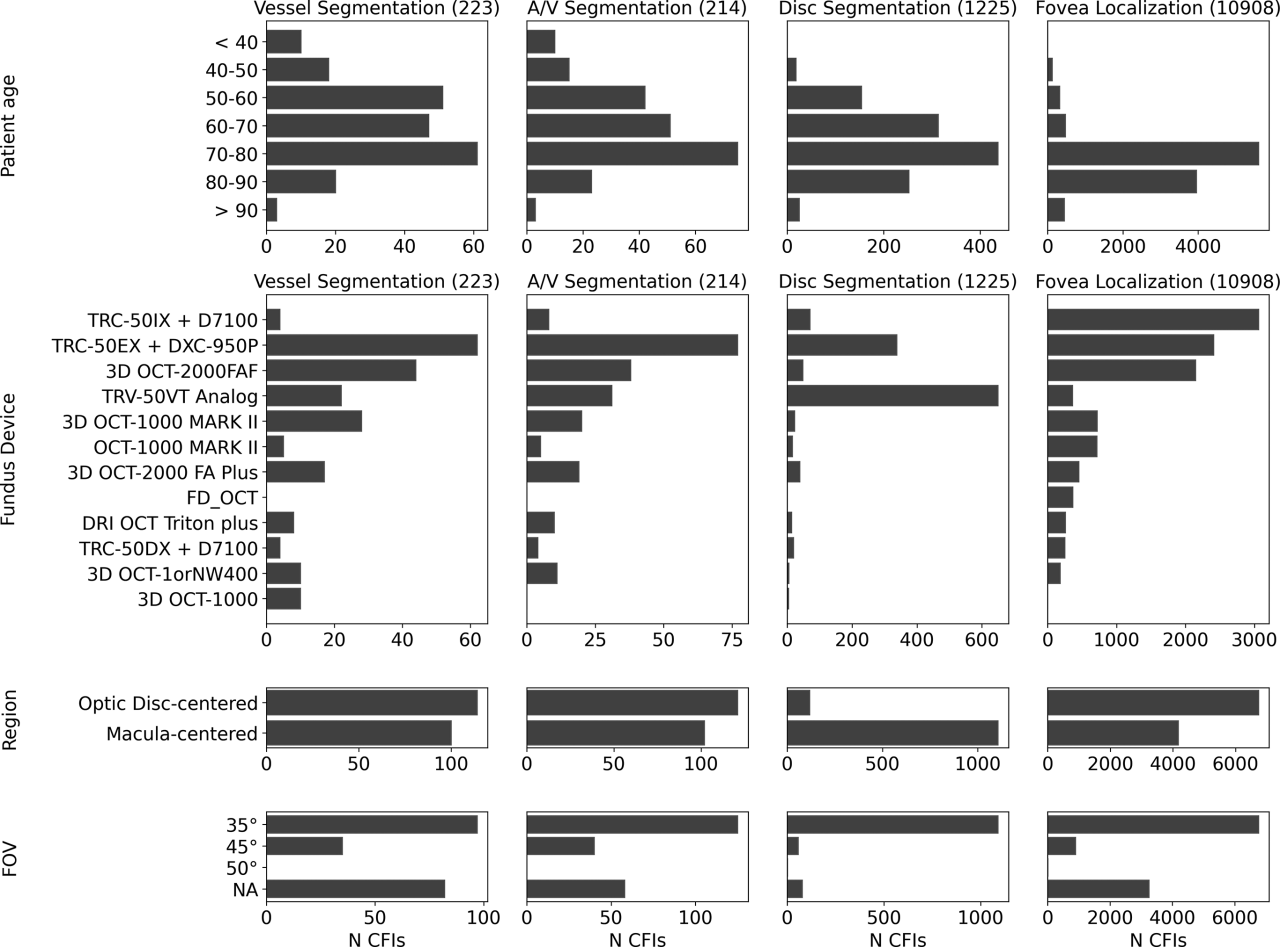
Statistics of the Rotterdam development sets used to train and evaluate VascX models. NA, not available.

For fovea detection, we used CFIs from a subcohort of the RS previously graded for signs of age-related macular degeneration, which included localization of the fovea for Early Treatment Diabetic Retinopathy Study (ETDRS) grid placement. We used the quality estimation model in AutoMorph^25^ to assess image quality and excluded images classified as *unusable.* Figure 1 shows the statistics of the final Rotterdam development sets. Vessel and artery-vein segmentation sets are similar (partly due to overlap) and closer to the overall distribution of the RS. The disc segmentation dataset, on the other hand, contains more images from the analog devices, which were often the hardest to segment, partly due to unconventional disc placement.

### CFI Annotation

**Figure 2.**
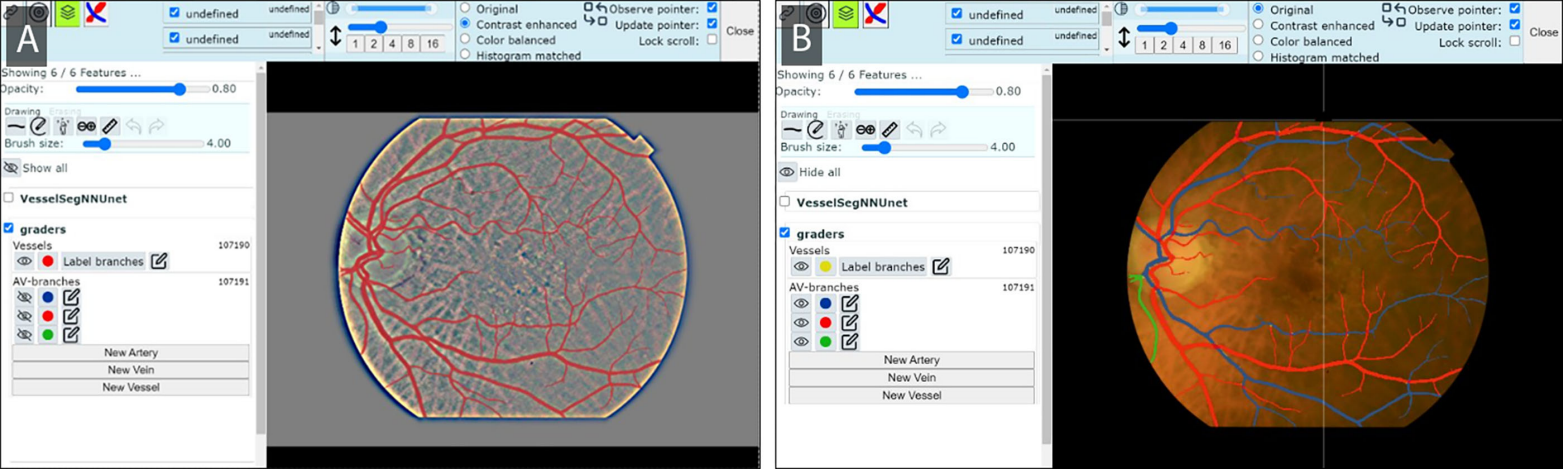
Screen captures of the software used for all the annotations on Rotterdam CFIs. (**A**) Vessel segmentation: graders corrected binary masks generated by artificial intelligence, using a digitally contrast-enhanced image to visualize vessels. (**B**) Artery-vein segmentation: graders colored the vessel masks into two separate (overlapping) masks for arteries and veins using a custom interface.

Images were annotated using custom software for ophthalmologic image annotation (Liefers B, et al. *IOVS*. 2024;65:ARVO E-Abstract 5891). Four professional graders participated in the process. The graders worked on desktop computers with a drawing tablet for segmentation. The annotation process was different per annotation type:

1. *Vessel segmentation*: We followed an artificial intelligence–assisted process by initializing the drawing interface with outputs from vessel segmentation models. Initially (first 200 images), we made use of models trained on public data (including AutoMorph^25^) to later switch to a model trained on both public data and annotations from previous iterations of this process. The graders were instructed to independently fix the masks produced by the model, including missing vessels and oversegmentation. A digitally enhanced version of the image with increased contrast of vessel edges was used to aid the graders (Fig. 2).
2. *Artery-vein segmentation*: We resolved connectivity issues at artery-vein crossings by annotating arteries and veins on separate layers. An interface was developed specifically for artery-vein segmentation (Fig. 2) to enable graders to start from a vessel segmentation (without A/V distinction) and color arteries and veins into two independent masks or layers using drawing tools. A third layer, *Unknown*, was added for vessels that could not be recognized as either of the other two. They were asked to color A/V crossings on both layers (overlapping). They were able to visualize and correct each layer independently or all at the same time. Another feature allowed them to visualize the connected components of each mask in different colors to find mistakes in connectivity easily. The entire process consisted of (1) correcting mistakes in the provided mask (vessel segmentation), (2) coloring the mask into A/V and unknown colors/layers, and (3) verifying connected components on the A/V masks and filling any gaps.
3. *Disc segmentation*: A single professional grader performed this annotation using the same drawing interface used for binary vessel segmentation (Fig. 2). The grader colored the entire optic disc area, excluding any peripapillary atrophy region, to match the annotations on public datasets.^41^^,^^46^^,^^53^ In case of doubt due to poor image quality or unclear disc boundaries, a consensus was reached among the three graders. Cases where the image quality was too poor to reach consensus were excluded.
4. *Fovea localization*: The fovea was localized by four graders who followed a standard grading protocol, which included placing an ETDRS grid over the images.

### Model Training

We trained our model ensembles on the datasets in Table 1. We implemented improved CFI bounds detection and preprocessing procedure and adapted the well-known NN–U-Net approach for model training.

#### Data Preprocessing

**Figure 3.**
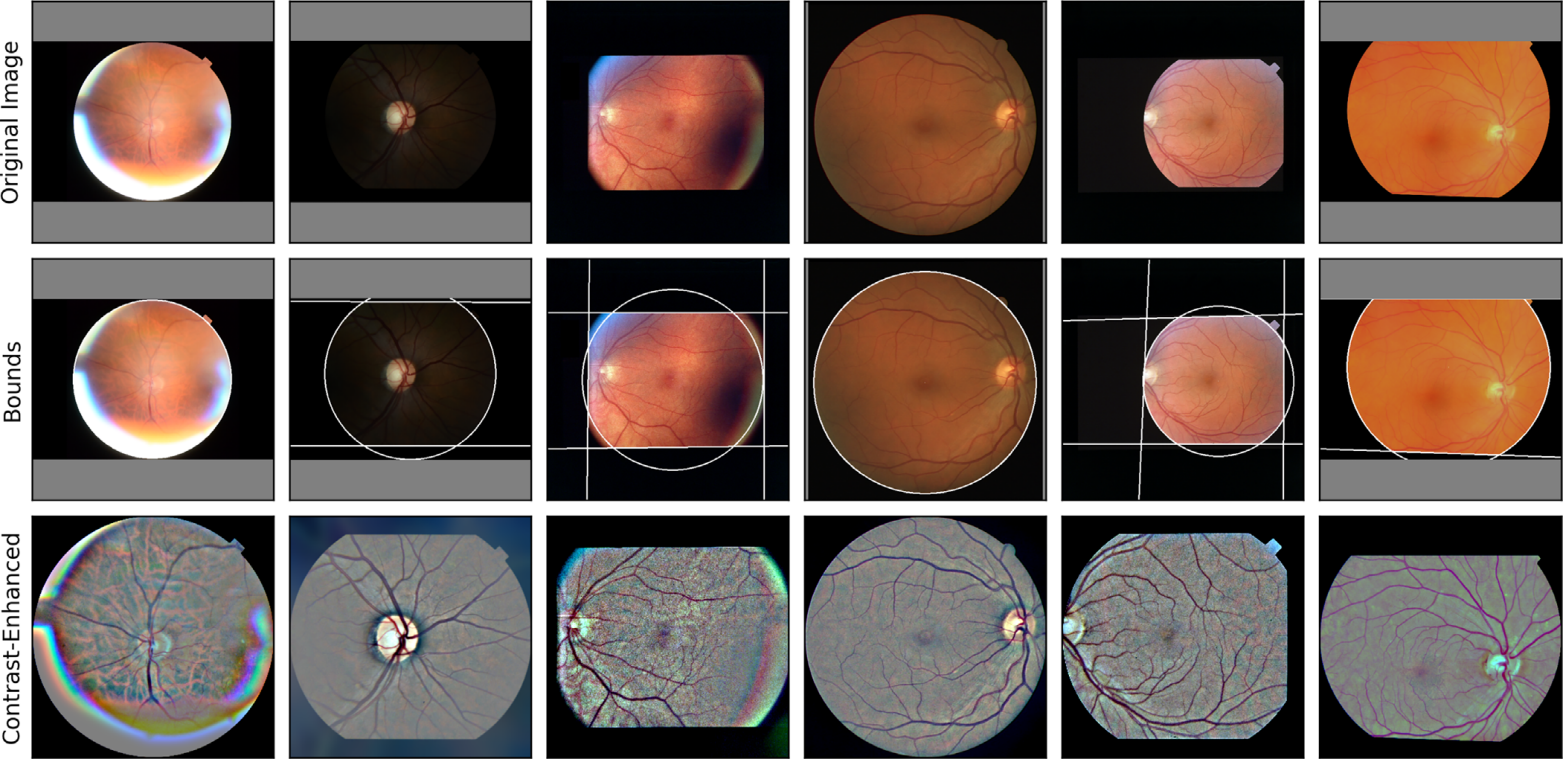
Sample of preprocessed images from the Rotterdam sets, showing: original images (*first row*), the results of bounds detection defined by the intersection of a circle and optional lines (*second row*), and the cropped and contrast-enhanced image (*third row*).

For all models, training and testing CFIs were preprocessed via:

1. Detection of the CFI boundaries defined by a circle and top, bottom, left, and right lines (see Fig. 3).
2. Cropping of the CFI along its boundaries into a square image and resizing to 1024 × 1024 px.
3. Contrast enhancement via Gaussian filtering. The image was mirrored along its boundaries before contrast enhancement to avoid artifacts, and the region outside its boundaries was blacked out after enhancement.

The geometric steps (cropping and resizing) were replicated on the labels for training. Figure 3 shows outputs of our preprocessing algorithm. Models were trained on the concatenation of the cropped CFI and its contrast-enhanced version (six input channels).

#### Vessel, A/V, and Disc Segmentation Models

Vessel and A/V segmentation models were trained separately, given the different development sets. Our training and inference pipeline was based on an open-source PyTorch implementation of NN–U-Net,^17^^,^^54^ including architecture, patching/windowing, and test-time augmentation. We added training augmentations specifically designed for fundus images to offset the relatively small size of A/V segmentation datasets. Training data were augmented via random appearance transforms: defocus, hue-saturation-value, and Gamma and Gaussian noise, followed by geometric transforms: flipping (horizontal and vertical), random scale (1.0–1.15 times original scale), random rotation (up to 10 degrees), and random elastic transforms. We used the Albumentations library^55^ for image and mask augmentation. Masks were interpolated via nearest-neighbor interpolation by the library.

Following the NN–U-Net approach, training and inference were done on patches of 512 × 512 px cropped out of a 1024 × 1024-px image. Patches were fed to a U-Net network with six input channels (for original RGB image and contrast-enhanced version), eight down-sampling stages, and deep supervision (loss calculated on the down-sampled stages too). Inference was done via a sliding window with 50% overlap and a Gaussian kernel for merging.

Vessel annotations were encoded as background (0) and vessel (1). Artery-vein annotations were encoded as background (0), arteries (1), veins (2), and crossings (both artery and vein) (3). We used this approach for A/V segmentation with the goal of boosting the relative importance of the crossings in the loss function. The loss function was the sum of Dice and cross-entropy with equal weights. The optic disc segmentation model was similarly trained on binary masks encoded as background (0) and disc mask (1), with the same loss function.

Supplementary Figure S6a shows sample training batches for the vessel and artery-vein segmentation models.

#### Fovea Localization Model

The fovea localization model was trained separately to localize keypoints on the CFIs. A heatmap regression approach^56^ with the same U-Net architecture was used for artery-vein segmentation as the backbone. Heatmap regression consists of training the model to regress a probability image or heatmap of the keypoint location. The pixel location of the maximum on the output heatmap is then taken as the output keypoint location. Train heatmaps may be generated using different probability density functions centered at the ground-truth keypoint location. We generated target heatmaps (512 × 512 px) using a Gaussian with constant *σ* centered at the keypoint. The value of *σ* = 50 px was selected via experiments on a holdout set for the task of localizing the disc center on CFIs. Mean squared error (MSE) was used as a loss function to train the model to approximate the continuous target heatmaps. The location (2D-index) of the maximum over the heatmaps output by the model was taken as the keypoint location.

Input CFIs were augmented via random appearance transforms: defocus, hue-saturation-value, and Gamma and Gaussian noise, followed by geometric transforms: horizontal flipping, random scale (1.0–1.05 times original scale), random rotation (up to 5 degrees), and finally resizing down to model input size. Test-time augmentation was correspondingly applied using the vertical flip of the input. Supplementary Figure S7 shows sample training batches for the disc segmentation and fovea localization models.

#### Quality Classification Model

The image quality classification model was trained using the same augmentations as for the fovea and disc models. The models were developed by fine-tuning ResNet-101 (ImageNet weights) with a 224-px input resolution. Here we used only the original RGB CFI (three channels; no contrast-enhanced version). All the model weights were fine-tuned (no frozen layers). Cross-entropy loss was used for classification and MSE for regression. Test-time augmentation was applied by averaging the outputs of the original and horizontally flipped inputs.

#### Training Setup

For all models, we made use of the *Albumentations* library for data augmentations. PyTorch and *pytorch-lightning* were used to develop our model training pipeline.

The batch size was set to 16 for all models. The *Adam* optimizer was used with *lr* = 0*.*001 for segmentation. Models were trained for a fixed number of epochs across folds: 100 for vessel segmentation, 200 for A/V segmentation, 100 for disc segmentation, and 35 for fovea localization. This was decided approximately based on experiments with a partition of 20% of the training set. We observed stable validation loss progression on these sets before and after the chosen epoch number and did not consider it necessary to do further optimization. Some architectural choices in NN–U-Net, such as the use of deep supervision and increased U-Net depth, were validated using the same partition and had only a marginal impact on Dice scores.

All models were trained on a single NVidia graphics card with 11 GB VRAM. Segmentation models were trained in mixed precision.

### Evaluation

We provided internal and external validation results for our models. For external validation, we employed a leave-one-dataset-out strategy, where we retrained our ensembles on all development datasets (Table 1), except one used for evaluation. This strategy allowed us to provide external validation results on various datasets, particularly the Rotterdam and Leuven-Haifa A/V segmentation datasets, which comprise most of our training set. Our goal is to provide an accurate picture of generalization performance across dataset characteristics.

For internal validation, we evaluated our models via fivefold cross-validation, with the Dice score as the main evaluation metric. We report the mean scores (separated per source dataset) over the five validation sets. Note that this evaluation setup means that the scores of our models could be slightly underrepresented when compared to the full ensemble of our models.

For all models, we input preprocessed images in 1024 × 1024-px resolution and used default inference settings.

To ensure a consistent comparison on the same images for all models, we evaluated entire datasets without automatic image quality filtering or other exclusions. For the A/V model, artery and vein masks were recovered from the model outputs. For the VascX model, this meant merging the *Crossings* output mask with both *Artery* and *Vein* masks.

## Results

### Comparison With Publicly Available Models

We compared the performance of our models with that of publicly available ones. Note that our goal was not to compare deep learning architectures but instead to compare the performance of trained models (including architecture, inference code, and public model weights) across different datasets (Table 1). Therefore, architectures published without publicly available weights have been excluded from this analysis.

We compared our three segmentation models to the model ensembles in AutoMorph,^25^ the most comparable pipeline that integrates vessel, A/V, and disc segmentation. Additionally, we compared vessel and A/V models to Little-WNet (LWNet),^20^ a dedicated vessel and A/V segmentation model with publicly available weights. Table 2 shows the results of external validation using Dice score to measure generalization performance to unseen datasets. The LWNet vessel segmentation weights were trained on DRIVE. For A/V segmentation, we tested both DRIVE and HRF-trained weights. We found DRIVE weights to perform better across all datasets and therefore report only these scores.

**Table 2.** Results of External Validation of Our Pipeline

*Vessel Segmentation*								
Model	DRHAGIS		FIVES		Leuven-Haifa		RS	
LWNet	0.672		0.756		0.713		0.745	
AutoMorph	0.741		0.824		0.812		0.810	
VascX	**0.750**		**0.832**		**0.829**		**0.844**	
*Artery-Vein Segmentation*								

	IOSTAR		AVRDB		Leuven-Haifa		RS	
Model	A	V	A	V	A	V	A	V

LWNet	0.326	0.551	0.390	0.588	0.569	0.649	0.635	0.696
AutoMorph	0.439	**0.621**	0.376	0.548	0.707	0.773	0.712	0.752
VascX	**0.455**	0.582	**0.463**	**0.598**	**0.778**	**0.822**	**0.790**	**0.821**
*Disc Segmentation*								

Model	IDRID		ORIGA		PAPILA		ADAM	

AutoMorph	0.931		0.876		0.618		0.958	
VascX	**0.963**		**0.940**		**0.959**		**0.965**	

VascX models were trained using a leave-one-dataset-out scheme and compared to the full ensemble of AutoMorph and LWNet models.

The best performance value for a particular dataset (column) is indicated in bold.

Vessel segmentation shows competitive performance between AutoMorph and VascX, with LWNet having the lowest performance. Differences between VascX and LWNet are not large, despite the larger training sets used for VascX. Performance across datasets is relatively consistent across all models, with DRHAGIS having slightly lower scores.

For A/V segmentation, VascX achieved higher Dice scores across most datasets, with higher performance for veins than for arteries, as observed in previous work.^21^ IOSTAR, where AutoMorph performed better for veins, was the only exception. Notably, some of the largest improvements are in the Rotterdam Study and Leuven-Haifa datasets, the most diverse and largest, respectively. Performance is less consistent across datasets, with scores on IOSTAR and AVRDB being lower than on Leuven-Haifa and Rotterdam datasets by a large margin.

Similarly, for optic disc segmentation, VascX reached mild but consistent improvements across most datasets when compared to AutoMorph. The largest was on the PAPILA dataset.

Table 3 shows the results of our internal VascX validation measuring generalization to unseen images from the same distributions used for training. VascX shows consistent performance across most datasets. Notably, differences between internal and external validation scores are not large for some datasets.

**Table 3.** Internal Validation of VascX Segmentation Models on Our Development Sets

*Vessel Segmentation (Dice Score)*												
Model	ChaseDB	DRHAGIS	HRF		RETA		FIVES		Leuven-Haifa		Rotterdam	
VascX	0.780	0.769	0.786		0.890		0.831		0.829		0.852	
*Artery-Vein Segmentation (Dice Score). A: Arteries Mask Score, V: Veins Mask Score*												

	HRF-AV		RITE		Les-AV		IOSTAR		Leuven-Haifa		Rotterdam	
Model	A	V	A	V	A	V	A	V	A	V	A	V

VascX	0.748	0.795	0.698	0.753	0.791	0.829	0.494	0.602	0.775	0.818	0.812	0.845
*Disc Segmentation (Dice Score)*												

Model	ORIGA	PAPILA	IDRiD		ADAM		PALM		REFUGE2		Rotterdam	

VascX	0.958	0.961	0.958		0.964		0.921		0.956		0.886	
*Fovea Localization (L2/Euclidean Distance to Ground Truth in Pixels)*												

Model	IDRID				ADAM		PALM		REFUGE2		Rotterdam	

VascX	14.68				11.15		26.22		10.68		25.14	

VascX models were trained and evaluated via five fold cross-validation. Scores are averages across folds.

Finally, our quality estimation model achieved an accuracy of 0.93, comparable to AutoMorph’s reported accuracy of 0.92,^25^ both for internal validation. Because we did not augment the EyeQ dataset (also used to train AutoMorph), we do not present further performance evaluation of this model.

**Figure 4.**
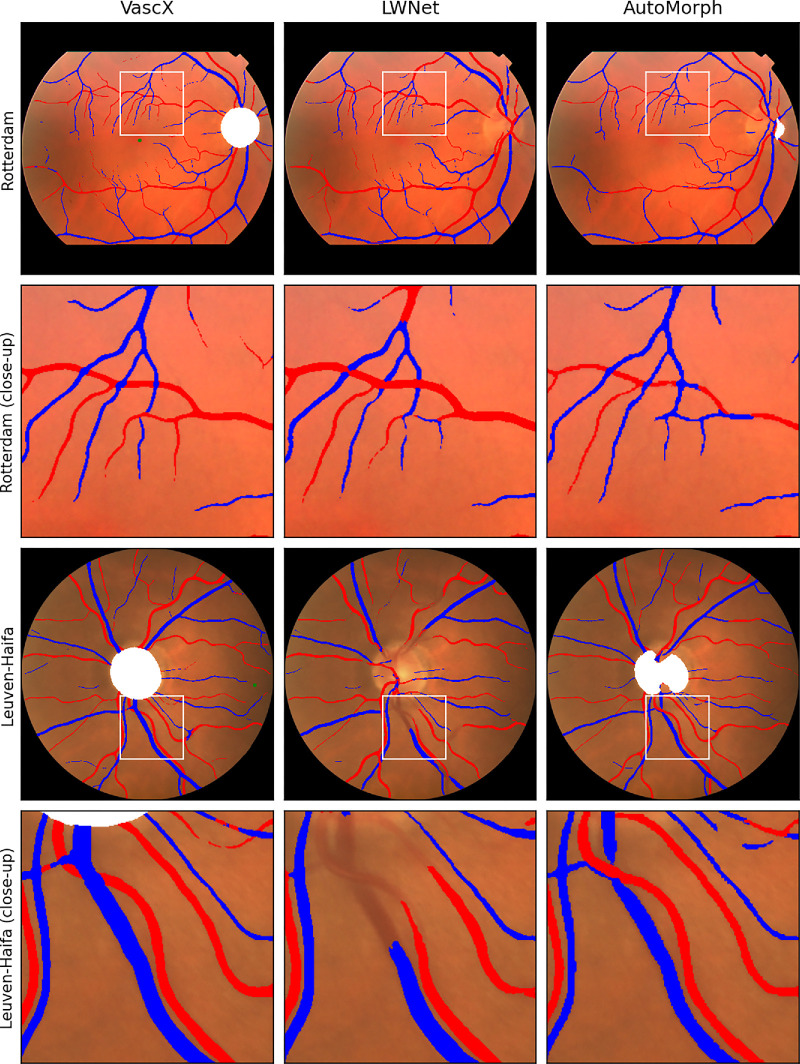
Example model outputs from the benchmarked systems showing A/V segmentation and disc and fovea outputs were applicable. We observed more consistency in A/V and disc segmentation from VascX. The CFIs shown correspond to the images with the median Dice score for A/V segmentation with VascX for each dataset.

Qualitatively, we observed a marked improvement in segmentation performance from VascX models. Figure 4 shows example model outputs from Rotterdam and Leuven-Haifa inputs (the two independent datasets). We observed more consistent disc detection from VascX, especially in cases where the disc was not clearly differentiated. A/V segmentation, while not flawless, produced visibly less continuity and fewer misclassification mistakes than both LWNet and AutoMorph. Importantly, many of the vessel crossings were correctly resolved on both artery and vein masks. More sample outputs for all segmentation models are shown in Supplementary Section B.

### Effect of Image Quality on Vessel and A/V Segmentation

**Figure 5.**
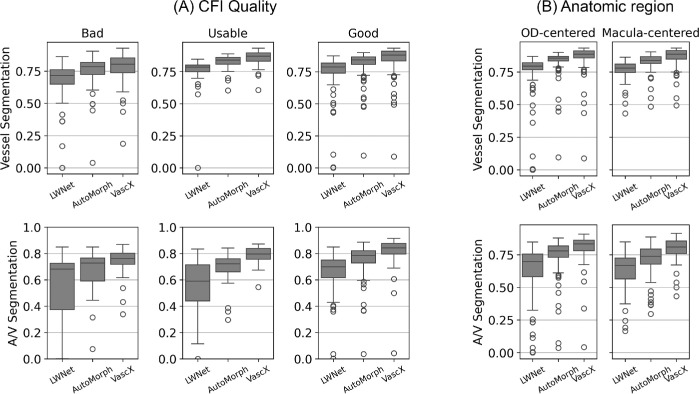
Boxplots displaying the distribution of model performance (Dice score) over image quality (**A**) and anatomic region (**B**) bins/slices of the Rotterdam dataset. For vessel segmentation, the performance of the models is stable across quality and anatomic region bins. For artery-vein segmentation, the biggest improvement is in usable images.

To more deeply characterize the performance of the models, we made use of the CFI quality estimation model to classify Rotterdam images into *good*, *usable*, and *bad/unusable* categories. We limited this analysis to the Rotterdam dataset due to the other dataset being smaller and/or highly uniform in terms of image quality and capture conditions. We used predictions from an ensemble trained on the rest of the datasets (external evaluation in Table 2). Figure 5 displays the distribution of Dice scores over image quality bins (as assessed by the models) and for optic disc and macula-centered images. Once again, vessel segmentation models achieved high consistency, even for CFIs classified as *bad* quality.

### Feature Accuracy

The primary goal of our models is to serve as the backbone for explainable vascular feature/biomarker extraction systems. Therefore, we quantified the quality of such features. We compared features extracted from model external evaluations (VascX, AutoMorph, and LWN) with reference features extracted from the annotations in our development set, which we considered ground truth. We quantified the quality of model features by measuring MAE and correlations with the ground-truth features. We used the Rotterdam set as the main evaluation set for this comparison due to its diversity and size. We evaluated the entire A/V development set without additional image selection (this set was originally filtered by the graders based on quality). We used predictions from the VascX A/V segmentation ensemble trained on datasets other than the Rotterdam Study (external evaluation in Table 2).

We made use of a vascular feature extraction pipeline to calculate a set of features, including tortuosity and CREs from segmentation masks. See Supplementary Section C.1 for feature implementation details. The features are calculated using artery-vein segmentation masks, and some (CREs, temporal angles, vascular density) use disc segmentation masks and fovea locations to define regions. Because LWNet does not include optic disc segmentation weights and AutoMorph struggled with the disc segmentation of Rotterdam images (Table 3), we used the same ground-truth disc masks in both pipelines. The fovea location was also the same across models. Therefore, this comparison evaluates the effect of different models for artery-vein segmentation only.

Table 4 shows the results of the comparison, where we measured reduced MAE and higher correlations when using VascX segmentations, across most features. Of 24 vascular features evaluated, 14 showed a significant improvement in MAE when compared to AutoMorph and 23 when compared to LWNet. VascX also had the highest correlations with ground-truth features in all but two cases.

**Table 4.** Comparison of Quality (MAE and Pearson *r*) of Features Extracted Using Different Models for Artery-Vein Segmentation: VascX, AutoMorph (AM), and LittleWNet (LWN) on the Rotterdam Sets (215 CFIs)

		MAE			Pearson *r*		
Characteristic	Grader μ	VascX	AM	LWN	VascX	AM	LWN
Temporal Angle—A	122.36	**6.16**	6.39^a^	8.63^b^	**0.69**	0.66	0.57
Temporal Angle—V	126.35	**3.83**	5.91	8.29^a^	**0.86**	0.73	0.65
CRE—A	10.53	**1.01**	1.49^b^	3.10^b^	**0.65**	0.57	0.49
CRE—V	15.83	**1.36**	1.94^b^	3.79^b^	**0.73**	0.70	0.51
Vasc. Density—A	4.31	**0.38**	0.87^b^	0.77^b^	**0.88**	0.76	0.61
Vasc. Density—V	4.97	**0.50**	0.89^b^	1.14^b^	**0.83**	0.63	0.60
Vessel Caliber [med]—A	4.23	0.67	**0.64**	1.36^b^	0.47	**0.48**	0.37
Vessel Caliber [std]—A	2.26	**0.27**	0.39^b^	0.90^b^	**0.68**	0.59	0.49
Vessel Caliber [med]—V	4.35	0.80	**0.72**	1.38^b^	0.49	**0.54**	0.38
Vessel Caliber [std]- V	3.15	**0.27**	0.47^b^	0.79^b^	**0.84**	0.69	0.64
Tortuosity [med]—A	1.09	**0.0057**	0.006	0.0059	**0.79**	0.73	0.76
Tortuosity [med]—V	1.09	**0.005**	0.0053	0.0059^a^	**0.69**	0.57	0.52
Curvature [med]—A	3.64	**0.45**	0.51^a^	0.54^a^	**0.92**	0.90	0.87
Curvature [med]—V	4.11	**0.48**	0.57^a^	0.58^a^	**0.87**	0.86	0.86
Inflection count [med]—A	1.46	**0.41**	0.61^b^	0.53^a^	**0.43**	0.26	0.30
Inflection count [med]—V	1.57	**0.46**	0.50	0.60^a^	**0.36**	0.32	0.26
Tortuosity [LW]—A	1.12	**0.015**	0.019^b^	0.038^b^	**0.59**	0.47	0.11
Tortuosity [LW]—V	1.11	**0.013**	0.014	0.033^b^	**0.64**	0.62	0.12
Bif. Angles [mean]—A	82.99	**6.64**	6.70	9.51^b^	**0.49**	0.41	0.27
Bif. Angles [med]—A	81.83	**5.35**	6.15^a^	8.97^b^	**0.57**	0.45	0.22
Bif. Angles [mean]—V	84.16	**5.30**	5.53	7.71^b^	**0.64**	0.59	0.54
Bif. Angles [med]—V	83.20	**4.60**	5.02	5.96^b^	**0.59**	0.52	0.56
Num. Bifurcations—A	27.32	**9.69**	10.97^a^	12.59^b^	**0.83**	0.77	0.69
Num. Bifurcations—V	31.61	**10.93**	12.06^a^	13.48^b^	**0.89**	0.76	0.76

Features extracted from ground-truth segmentations were used as reference. The feature implementation was the same across models. For vessel or bifurcation features, the aggregation function (applied on features from the whole image) is indicated in brackets: [med], median; [std], standard deviation; [mean], mean value; [LW], length-weighted; vessels were weighted by their length. Grader μ is the mean of feature values; mean MAE is the mean absolute error between ground-truth features and features extracted from model outputs. Pearson coefficients are correlations between ground-truth and model outputs.

The lowest MAE and highest Pearson r per feature (row) is indicated in bold.

a Significant difference in MAE (*P <* 0*.*05) between the model and VascX.

b *P <* 0*.*001.

We report results of the same comparison on the Leuven-Haifa dataset (240 images with 30 fields of view) in Supplementary Table S6. These results show a similar behavior. Although fewer features improved over AutoMorph, most error differences and correlations favored VascX.

## Discussion

We presented new model ensembles for CFI vasculature analysis and benchmarked them against publicly available systems. With the objective of developing models that operate robustly across devices and achieve performance improvements on the different imaging conditions found in real cohort studies and clinical datasets, we focused on the diversity of our development set. We augmented more than 15 public datasets (each of which usually consists of carefully selected CFIs from one or a few devices) with CFIs collected in The Netherlands throughout more than three decades from 11 different imaging devices and with diverse pathology. Together, this resulted in a comprehensive set of images, including a wider range of patient characteristics (origin, age, pathologies) and capture characteristics (device, anatomic region, field of view). For model training, we made use of well-known architectures, a new and more robust preprocessing algorithm, and data augmentation to achieve solid performance across tasks.

Our main evaluation using Dice scores revealed consistently better performance from VascX when compared to existing publicly available models—AutoMorph and LWNET—across both public datasets and the Rotterdam sets. We observed an important improvement in A/V segmentation performance, which is crucial for vascular analysis pipelines. We observed a lower performance across models in the IOSTAR and AVRDB datasets when compared to RS and Leuven-Haifa. This is caused partly by the oversegmentation of small vessels in the ground truth of IOSTAR and AVRDB. This can be seen in Supplementary Figures S9 and S10, where the ground-truth masks are considerably thicker than model predictions. Close inspection of these datasets reveals that the ground-truth segmentations are rough and often extend beyond vessel boundaries. It stands out that the largest difference between VascX and the rest is in *usable* or intermediate-quality images, which are common in large cohort studies and clinical data.

Consistent improvements were also observed in the disc segmentation task. A large difference was also observed in PAPILA 2, which has a 30-degree field of view not present in AutoMorph’s training set. AutoMorph failed especially in cases in which the optic disc border did not exhibit a clear contrast difference, as seen in Supplementary Figure S11.

The differences in vessel segmentation performance were smaller between models. This suggests that the size of vessel segmentation datasets has reached a point where further improvements will be increasingly difficult with current architectures.

Our fovea localization model had mean L2 errors between 10.68 (REFUGE2) and 26.22 (PALM) on images of 1024 × 1024 px. This model is enough to provide an approximate localization of the fovea center, useful for the automatic placement of retinal regions (e.g., ETDRS grid) for localized measurements.

Importantly, our results showed that better segmentation performance translates into more accurate vascular features, in line with the results of Fhima et al.^21^ Features from VascX model segmentations show lower absolute error and higher correlation with features extracted directly from grader segmentations, when compared to features extracted from AutoMorph segmentations.

Further work is required in several areas. There is room for improvement in artery-vein segmentation, especially regarding the connectivity of the vessel trees. Despite the improvements, our model still produces A/V misclassifications and gaps. Enforcing the expected tree structure in the segmentation is a notorious open challenge in deep learning. Incorporating specific loss functions^57^ and postprocessing steps^58^ designed for connectivity may help address this issue.

The further development of retinal analysis pipelines, as well as the development and evaluation of vascular features with clinical significance, also holds promise. The feature computation stage involves significant nuance. Some features, such as tortuosity, may be particularly sensitive to discontinuities in the segmentation. The results in Table 4 support that some features are more robust than others to potential mistakes in the segmentations. Deeper evaluations of the robustness and predictive power of vascular features are in order. The ability to capture image quality for quality control and for informing feature computation may be crucial here. Classification of image quality using a one-dimensional image-level label, while useful, is not granular enough for some applications. Artifacts may affect only part of the image. Defocus or poor overall quality may result in a CFI usable for analysis of the main vascular arches but not any smaller vessels. A decomposition of image quality into (localized) factors may be a necessary next step in developing more robust pipelines.

In summary, our results show that our models outperform those in previous systems in every dataset and condition we evaluated, likely due to the increased size and variety of our training set. We expect that VascX models will serve as the backbone for more robust CFI vascular analysis pipelines (and other CFI-based pipelines). To serve these goals, we have made available our entire inference pipeline, including model weights and inference code with a new CFI preprocessing algorithm. With this, we aim to catalyze research toward understanding how analysis of the retinal vasculature can help us prevent, detect, and monitor diseases, not only of the eye but of the rest of the body.

## Supplementary Material

Supplement 1

Supplement 2

Supplement 3

Supplement 4

Supplement 5

Supplement 6

Supplement 7

Supplement 8

Supplement 9
