# Supplementary material for: VascX Models: Deep Ensembles for Retinal Vascular Analysis From Color Fundus Images
Source: Transl Vis Sci Technol. 2025 Jul 23;14(7):19. doi: 10.1167/tvst.14.7.19 (PMC12306690; doi:10.1167/tvst.14.7.19)
Supplement: Supplement 1 [file tvst-14-7-19_s001.pdf]

Sample Training Batches

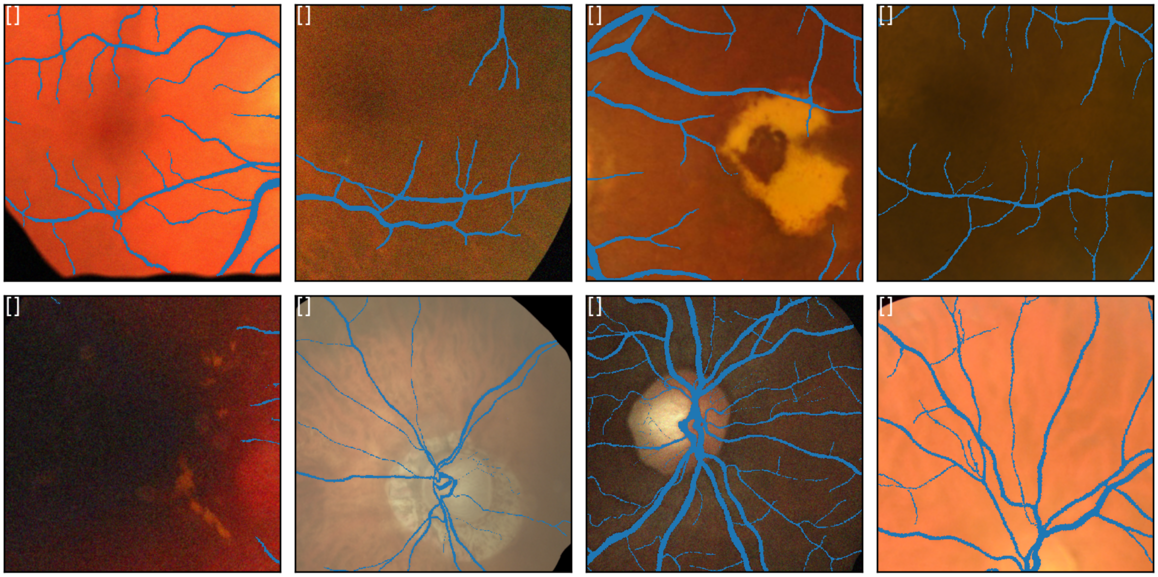

(a) Vessel segmentation.

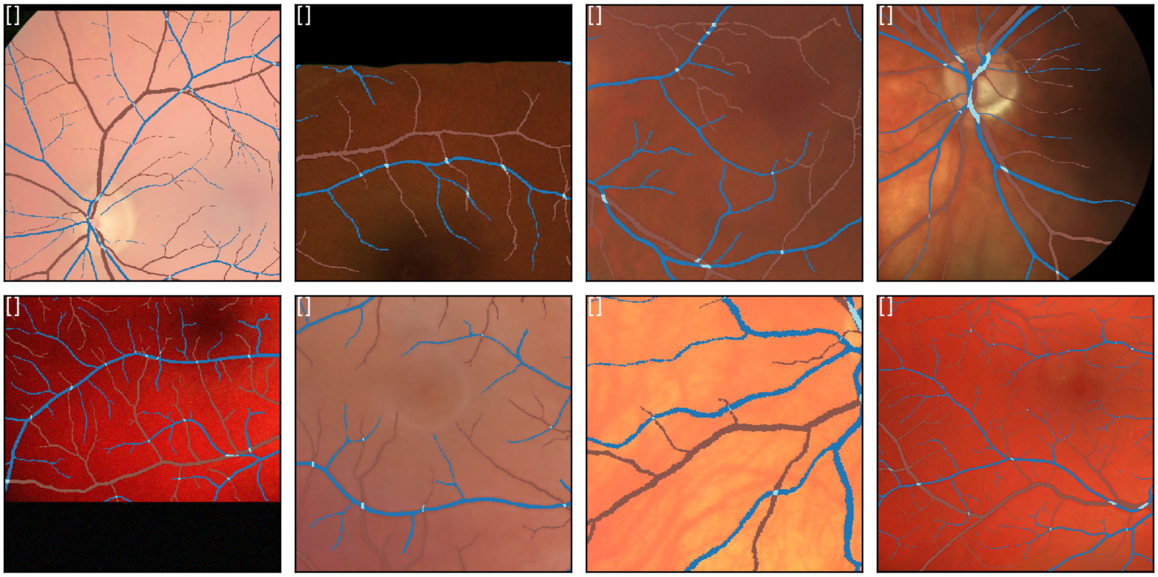

(b) Artery-vein segmentation, where arteries, veins and crossings are separate (exclusive) classes.

Figure 6. Sample training batches of input images to vessel and artery-vein segmentation models after data augmentation, including the CFI and overlaid masks. The contrast enhanced image is not shown.
