# Supplementary material for: VascX Models: Deep Ensembles for Retinal Vascular Analysis From Color Fundus Images
Source: Transl Vis Sci Technol. 2025 Jul 23;14(7):19. doi: 10.1167/tvst.14.7.19 (PMC12306690; doi:10.1167/tvst.14.7.19)
Supplement: Supplement 2 [file tvst-14-7-19_s002.pdf]

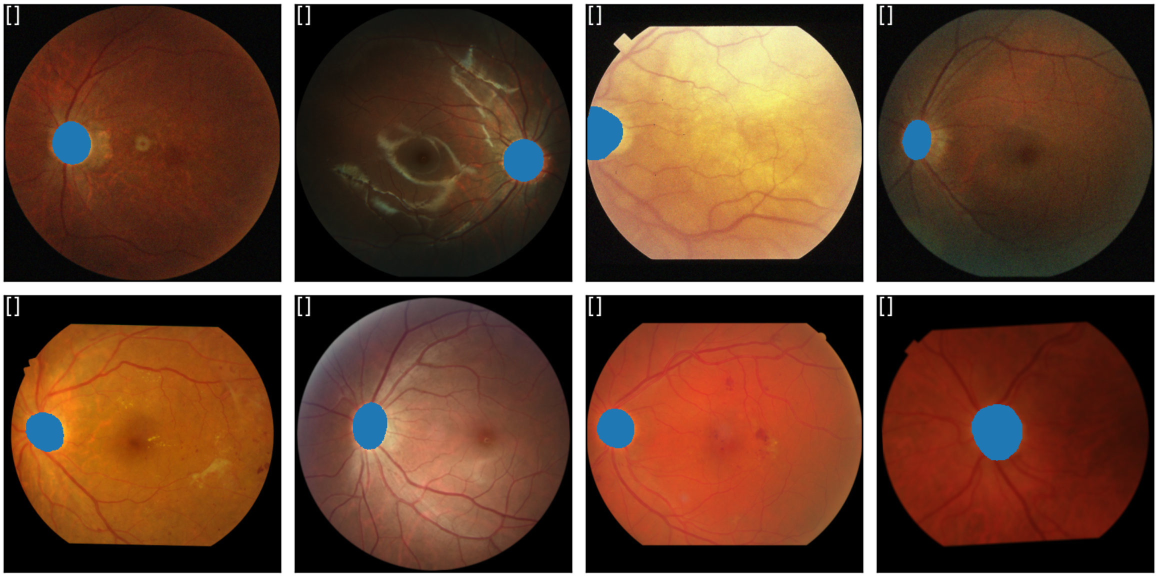

(a) Disc segmentation masks.

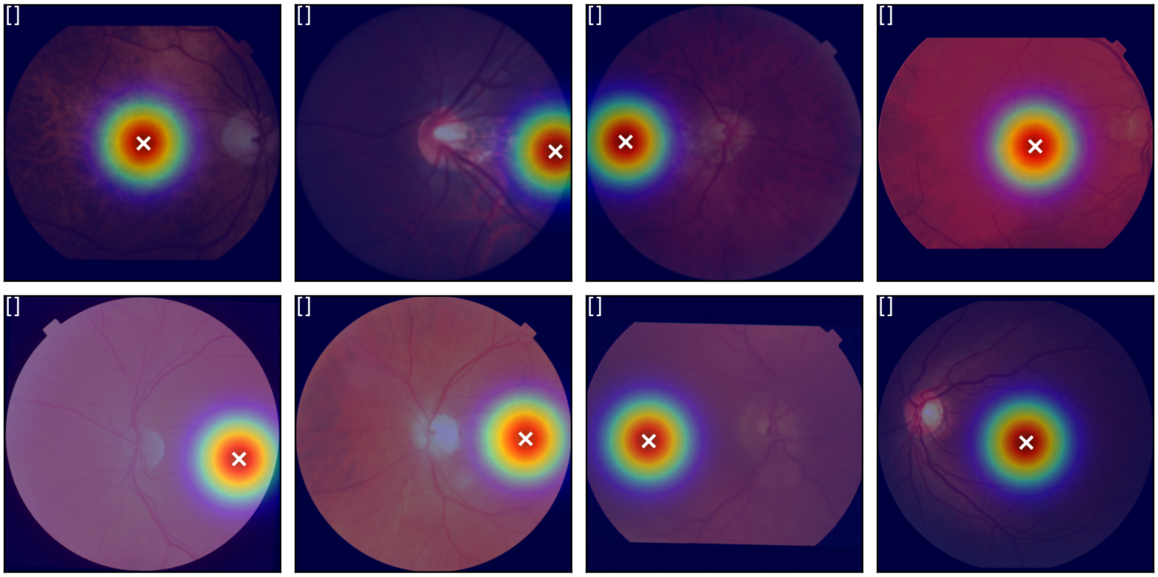

(b) Fovea localization heatmaps overlaid over the input images.

Figure 7. Sample training batches of input images to disc segmentation and fovea localization models after data augmentation. The contrast enhanced image is not shown.
