# Supplementary material for: VascX Models: Deep Ensembles for Retinal Vascular Analysis From Color Fundus Images
Source: Transl Vis Sci Technol. 2025 Jul 23;14(7):19. doi: 10.1167/tvst.14.7.19 (PMC12306690; doi:10.1167/tvst.14.7.19)
Supplement: Supplement 4 [file tvst-14-7-19_s004.pdf]

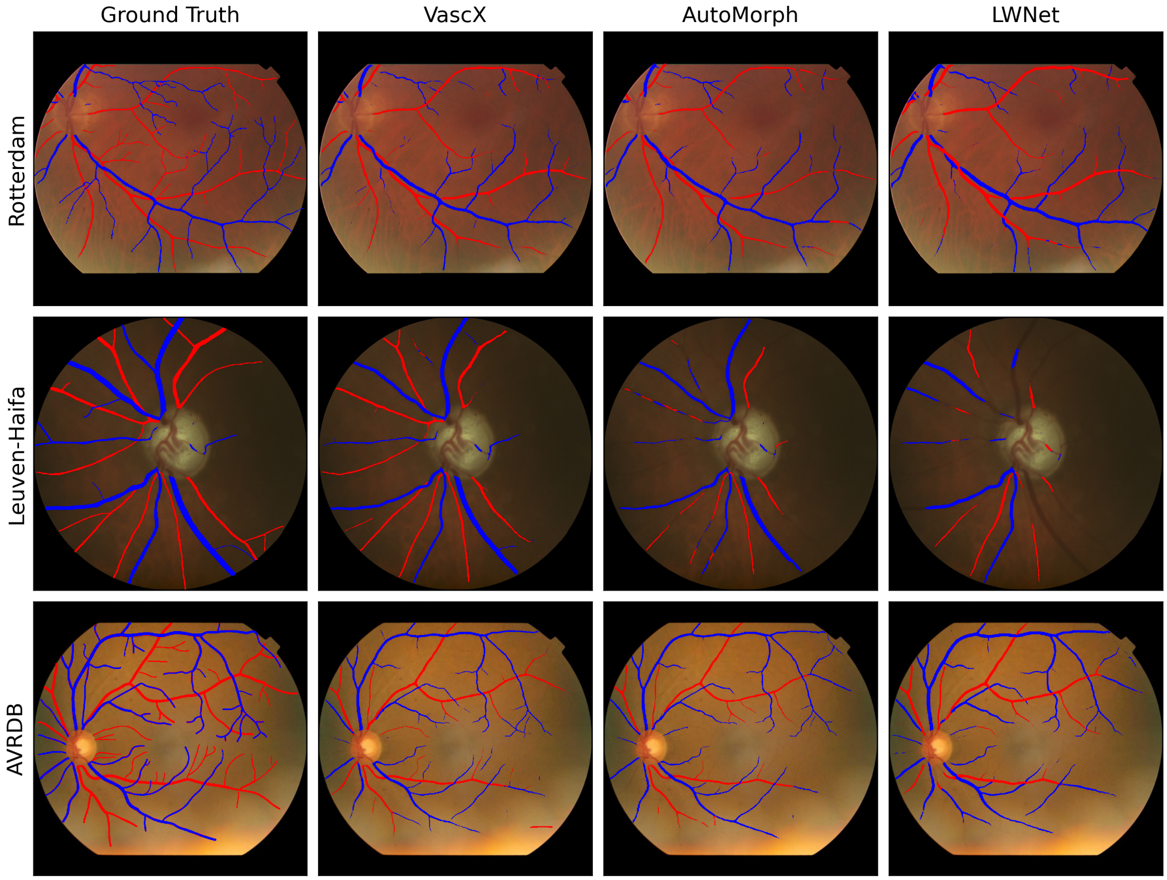

Figure 10. Sample artery-vein segmentation outputs from VascX, Automorph and LWNet for images classified as *bad* quality.
