# Supplementary figures and images for: VascX Models: Deep Ensembles for Retinal Vascular Analysis From Color Fundus Images
Source: Transl Vis Sci Technol. 2025 Jul 23;14(7):19. doi: 10.1167/tvst.14.7.19 (PMC12306690; doi:10.1167/tvst.14.7.19)

### B.3 Disc segmentation

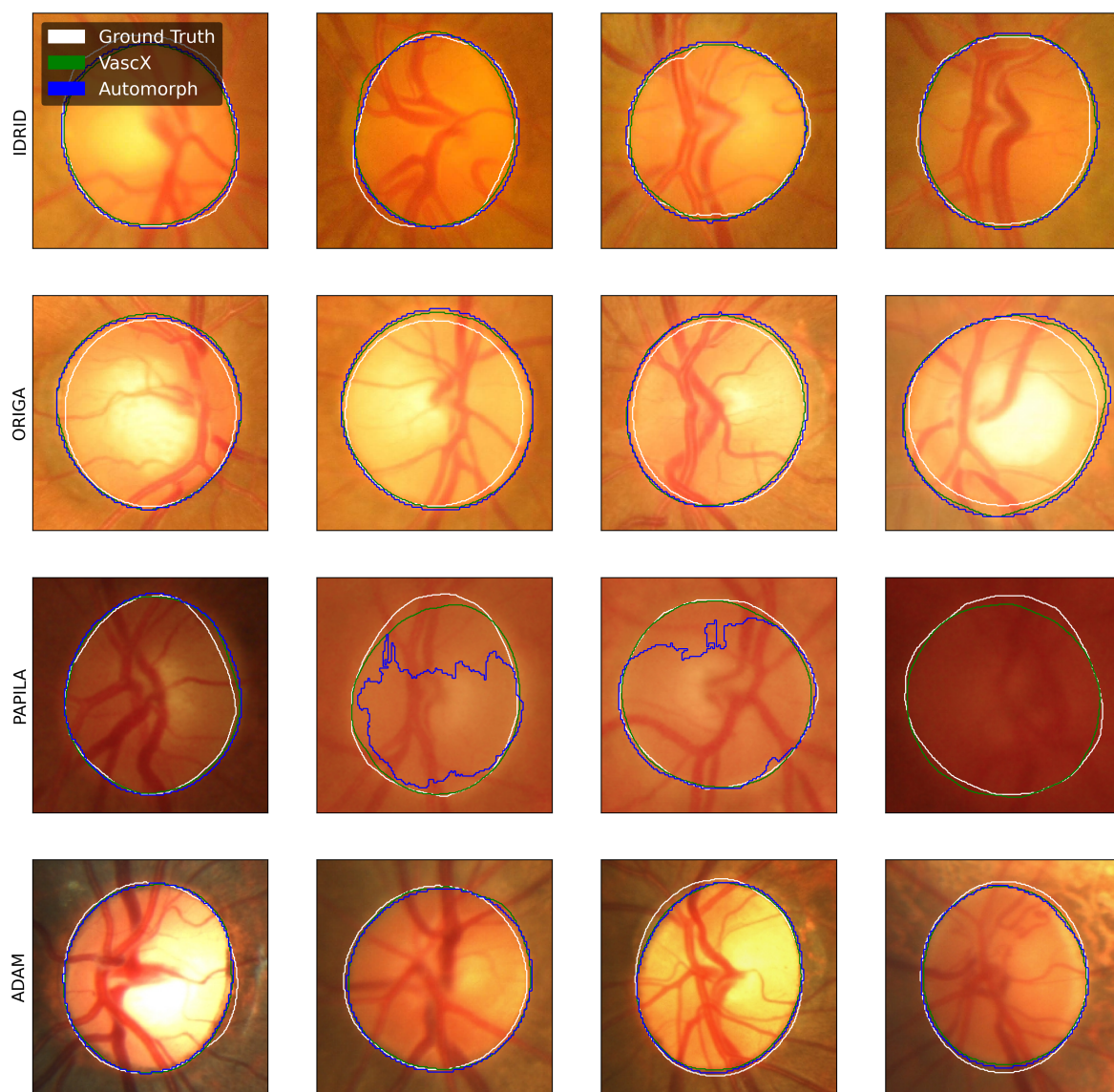

**Figure 11.** Sample disc segmentation outputs from VascX and Automorph.

Supplement: Supplement 5 [file tvst-14-7-19_s005.pdf]
