# Supplementary material for: VascX Models: Deep Ensembles for Retinal Vascular Analysis From Color Fundus Images
Source: Transl Vis Sci Technol. 2025 Jul 23;14(7):19. doi: 10.1167/tvst.14.7.19 (PMC12306690; doi:10.1167/tvst.14.7.19)
Supplement: Supplement 6 [file tvst-14-7-19_s006.pdf]

A Dataset Details

Table 5. Complementary details of the public dataset used in model development. *: 379 of 1200 samples were discarded due to containing no disc segmentation for partially visible discs. †: 21 of 1200 CFIs were discarded due to having no associated annotation.

Dataset	N	Resolution	Devices
**Vessel Segmentation**			
Chase DB[32]	28	1280×960	Nidek NM-200-D
DRHAGIS[33]	40	$4752 \times 3168 - 2816 \times 1880$	Topcon TRC-NW6s, Topcon TRC-NW8, Canon CR DGi
HRF[31]	45	3504×2336	NR
RETA[34]	54	4288×2848	Kowa VX-10 alpha
FIVES[35]	800	2048×2048	Topcon TRC-NW8
Leuven-Haifa[30]	240	1444×1444	Zeiss Visucam 500
Rotterdam (ours)	352	see Fig. 1	
**Total**	**1559**		
**Artery Vein Segmentation**			
RITE[36]	40	768×584	Canon CR5 3CCD
HRF-AV[37]	45	3504×2336	NR
Les-AV[38]	22	1444×1620	NR
IOSTAR[39]	30	1024×1024	i-Optics EasyScan
AVRDB[40]	100	1504×1000	Topcon TRC-NW8
Leuven-Haifa[30]	240	1444×1444	Zeiss Visucam 500
Rotterdam (ours)	215	see Fig. 1	
**Total**	**562**		
**Disc Segmentation**			
ORIGA[41]	650	NR	NR
PAPILA[42]	488	2576×1934	Topcon TRC-NW400
IDRiD[43]	81	4288×2848	Kowa VX-10 alpha
ADAM[44]	821*	2124×2056, 1444×1444	Zeiss Visucam 500, Canon CR-2
PALM[45]	1179†	2124×2056, 1444×1444	Zeiss Visucam 500, Canon CR-2
REFUGE2[46]	2000	1634×1634 - 2124×2056	Zeiss Visucam 500, Canon CR-2, Topcon TRC-NW400, Kowa
Rotterdam (ours)	1225	see Fig. 1	
**Total**	**7464**		
**Fovea Localization**			
IDRiD[43]	516	4288×2848	Kowa VX-10 alpha
ADAM[44]	1200	2124×2056, 1444×1444	Zeiss Visucam 500, Canon CR-2
PALM[45]	1200	2124×2056, 1444×1444	Zeiss Visucam 500, Canon CR-2
REFUGE2[46]	2000	1634×1634 - 2124×2056	Zeiss Visucam 500, Canon CR-2, Topcon TRC-NW400, Kowa
Rotterdam (ours)	10908	see Fig. 1	
**Total**	**15824**		
