# Supplementary material for: VascX Models: Deep Ensembles for Retinal Vascular Analysis From Color Fundus Images
Source: Transl Vis Sci Technol. 2025 Jul 23;14(7):19. doi: 10.1167/tvst.14.7.19 (PMC12306690; doi:10.1167/tvst.14.7.19)
Supplement: Supplement 7 [file tvst-14-7-19_s007.pdf]

C.2 Feature Quality Comparison

Additional feature quality comparison tables between VascX, Automorph and LWNet, complementing the Results section of the main paper.

	Grader μ	MAE			Pearson r		
		VascX	AM	LWN	VascX	AM	LWN
Temporal Angle - A	127.67	**4.17**	5.16**	11.25**	**0.78**	0.76	0.46
Temporal Angle - V	132.64	**3.02**	4.66	12.16**	**0.87**	0.75	0.37
CRE - A	12.21	**1.63**	1.97*	3.03**	**0.61**	0.38	0.26
CRE - V	19.89	**1.94**	2.85**	4.71**	**0.72**	0.57	0.14
Vessel Caliber [med] - A	5.31	**1.28**	1.35	1.70**	**0.30**	0.22	0.13
Vessel Caliber [std] - A	3.00	**0.41**	0.63**	0.66**	**0.71**	0.56	0.47
Vessel Caliber [med] - V	5.48	**1.46**	1.58	2.17**	**0.48**	0.34	0.21
Vessel Caliber [std]- V	4.76	**0.45**	0.90**	0.75**	**0.79**	0.66	0.34
Vasc. Density - A	4.76	**0.61**	1.19**	1.49**	**0.74**	0.60	0.44
Vasc. Density - V	5.44	**0.64**	1.02**	1.31**	**0.78**	0.65	0.40
Tortuosity [med] - A	1.08	**0.005**	0.0064*	0.0076**	**0.78**	0.74	0.68
Tortuosity [med] - V	1.08	**0.0045**	0.0048	0.0065**	0.75	**0.77**	0.51
Curvature [med] - A	2.84	0.50	**0.48**	0.62*	0.89	**0.90**	0.84
Curvature [med] - V	3.42	0.57	**0.56**	0.59	**0.85**	0.82	0.81
Inflection count [med] - A	1.70	0.59	**0.58**	0.71*	0.08	0.15	**0.17**
Inflection count [med] - V	1.90	**0.62**	0.73	0.76*	**0.37**	0.13	0.29
Tortuosity [LW] - A	1.11	**0.022**	0.026**	0.068**	0.15	**0.16**	-0.31
Tortuosity [LW] - V	1.11	**0.018**	0.022**	0.06**	**0.38**	0.25	0.02
Bif. Angles [mean] - A	79.59	7.84	**7.03**	11.62**	**0.65**	0.55	0.32
Bif. Angles [med] - A	78.31	7.44	**7.08**	10.99**	**0.65**	0.52	0.33
Bif. Angles [mean] - V	81.19	**6.82**	7.03	10.17*	**0.53**	0.39	0.24
Bif. Angles [med] - V	81.48	**6.68**	6.78	9.50**	**0.56**	0.42	0.29
Num. Bifurcations - A	15.41	**4.69**	6.69**	7.44**	**0.71**	0.69	0.63
Num. Bifurcations - V	17.43	**6.46**	7.49**	8.93**	**0.76**	0.70	0.55

Table 6. Comparison of quality (MAE and Pearson r) of features extracted using different models for artery-vein segmentation: VascX, Automorph (AM) and LittleWNet (LWN) on the Leuven-Haifa dataset (240 CFIs). Features extracted from ground truth segmentations were used as reference. For vessel or bifurcation features, the aggregation function (applied on features from the whole image) is indicated in brackes: [med]: median, [std]: standard deviation, [mean]: mean value, [LW]: length-weighted; vessels were weighted by their length. The feature implementation was the same across models. Grader μ is the mean of feature values; mean MAE is the mean absolute error between ground truth features and features extracted from model outputs. Pearson coefficients are correlations between ground truth and model outputs. *: Significant difference in MAE ($p < 0.05$) between the model and VascX. †: $p < 0.001$
