# Supplementary material for: VascX Models: Deep Ensembles for Retinal Vascular Analysis From Color Fundus Images
Source: Transl Vis Sci Technol. 2025 Jul 23;14(7):19. doi: 10.1167/tvst.14.7.19 (PMC12306690; doi:10.1167/tvst.14.7.19)
Supplement: Supplement 8 [file tvst-14-7-19_s008.pdf]

B Sample Outputs

B.1 Vessel segmentation

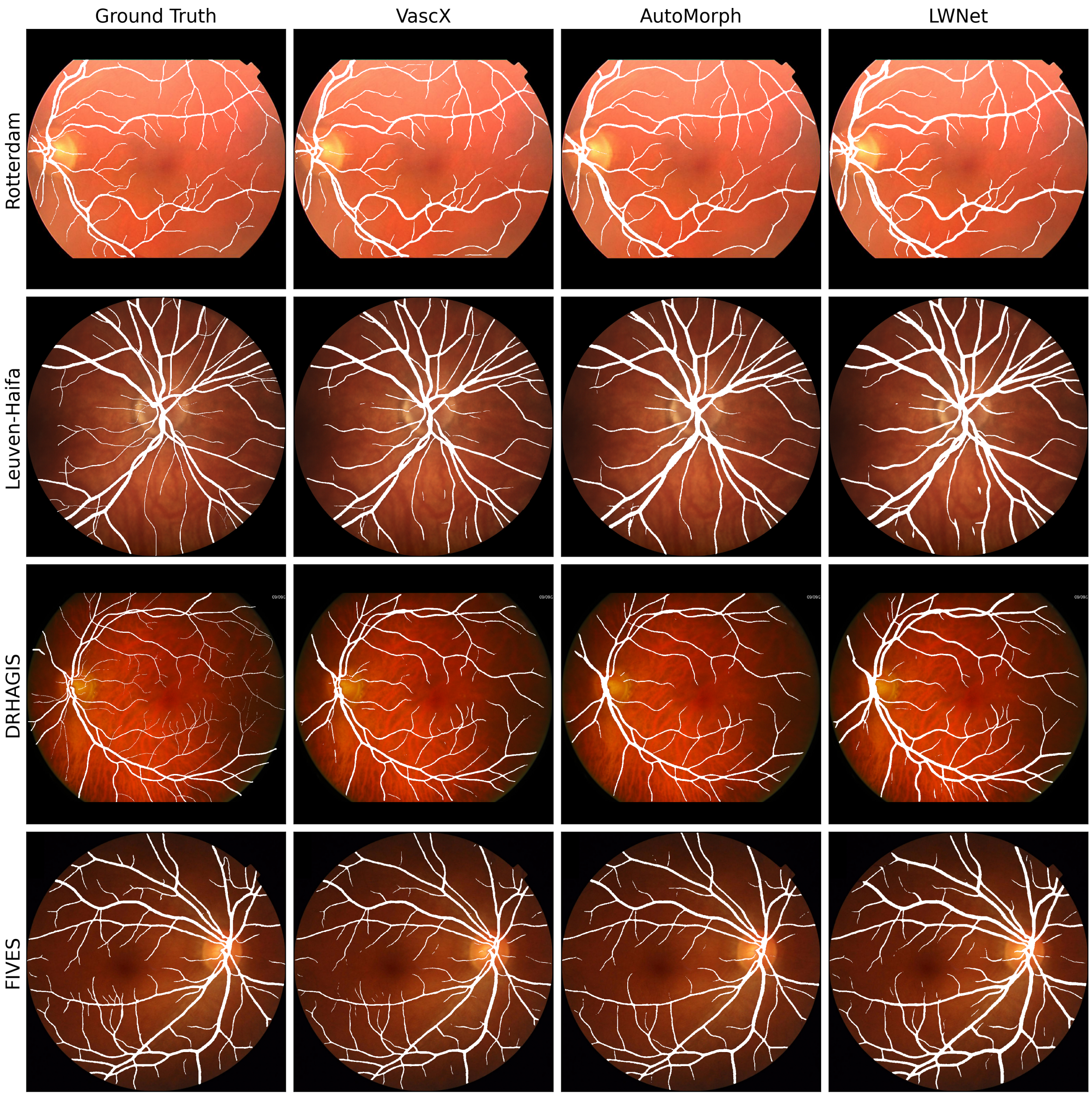

Figure 8. Sample vessel segmentation outputs from VascX, Automorph and LWNet.
