# Supplementary material for: VascX Models: Deep Ensembles for Retinal Vascular Analysis From Color Fundus Images
Source: Transl Vis Sci Technol. 2025 Jul 23;14(7):19. doi: 10.1167/tvst.14.7.19 (PMC12306690; doi:10.1167/tvst.14.7.19)
Supplement: Supplement 9 [file tvst-14-7-19_s009.pdf]

C Feature Comparison

C.1 Feature Implementations

In this section we describe the implementation of our vascular features, or refer to previous work for a detailed description.

Temporal Angle We followed the implementation presented in[59] (Section 2.3 of Supplementary Material).

CRE We followed the implementation presented in[59] (Section 2.2 of Supplementary Material). However, we measure and aggregate all vessels on the temporal side of the image that intersect the defined circle. We use only the temporal side because the nasal side is often not visible at the evaluated radii in fovea-centered images.

Vascular Density : Defined as the fraction of 1-pixels in the binary A/V segmentation mask within the fundus mask area:

$$\text{Vascular Density} = \frac{\sum_{i=1}^{H} \sum_{j=1}^{W} I(i,j) \cdot M(i,j)}{\sum_{i=1}^{H} \sum_{j=1}^{W} M(i,j)}$$

where:

- $I(i,j)$ is the binary vessel mask where each pixel is either 0 (background) or 1 (vessel).
- $M(i,j)$ is the binary mask where the region of interest (ROI) is defined, as extracted by our pre-processing algorithm

Vessel Caliber Vessel calibers or widths were measured per vessel segment by first fitting a spline to the segment skeleton. Perpendiculars to the spline were sampled at regular intervals. A ray casting method was used to extend the lines until they reached a background pixel (in the original artery or vein mask) on both sides, resulting in a width measurement. The median of the measurements was taken as the vessel segment caliber.

Tortuosity Tortuosity was defined using its common definition as the length of the vessel following its points divided by its chord length (distance between its endpoints):

$$T = \frac{\sum_{i=1}^{N-1} \sqrt{(x_{i+1} - x_i)^2 + (y_{i+1} - y_i)^2}}{\sqrt{(x_N - x_1)^2 + (y_N - y_1)^2}}$$

where (x_i, y_i) are the coordinates of the skeleton points, with $i = 1, 2, ..., N$ where N is the total number of points along the vessel skeleton.

Curvature Curvature is an alternative measure of tortuosity. Given a parametrization $\gamma(t) = (x(t), y(t))$ of a twice differentiable plane curve, curvature can be expressed as:

$$\kappa = \frac{|x'y'' - y'x''|}{(x'^2 + y'^2)^{3/2}}$$

where the derivatives x', y', x'' and y'' are with respect to t.

The mean curvature over the curve can be defined by integration of this expression:

$$\bar{\kappa} = \int_0^1 \kappa(t)$$

This calculation was approximated by computing κ at regular intervals (every 5 pixels), using splines as parametrization to approximate derivatives.

Inflection count Another alternative measure of tortuosity, this feature counts the number of inflection points (changes of direction) in a vessel segment.

Num. Bifurcations The number of bifurcations detected in the image. Bifurcations are detected by converting the skeleton into a graph, which is then traversed using the optic disc location as reference to defined outgoing vessels.

Bif. Angles Smallest angle between the two child vessels in a bifurcation. A distance δ from the bifurcation point was defined, at which the angle is measured. The spline approximations are used to obtain points (x, y) along the vessel segments at such δ. Bifurcations with child vessels shorter than δ are ignored.
